# Viscoelastic and shear-thinning effects of aqueous exopolymer solution on disk and sphere settling

**DOI:** 10.1038/s41598-019-44233-z

**Published:** 2019-05-27

**Authors:** Magdalena M. Mrokowska, Anna Krztoń-Maziopa

**Affiliations:** 1 Institute of Geophysics, Polish Academy of Sciences, Ks. Janusza 64, 01-452 Warsaw, Poland; 2 Warsaw University of Technology, Faculty of Chemistry, Noakowskiego St. 3, 00-664 Warsaw, Poland

**Keywords:** Limnology, Ocean sciences, Chemical physics, Fluid dynamics

## Abstract

In this study, xanthan gum is used as a model exopolymer to demonstrate potential effects of non-Newtonian properties of natural aquatic systems on settling dynamics of particles. Rheological measurements combined with settling experiments using visualization methods revealed that instantaneous velocity fluctuations and a flow pattern formed around a particle are the effects of solution viscoelasticity and shear-thinning properties and that the average settling velocity depends on the exopolymer concentration and particle size. Our study showed that in the considered conditions a disk-shaped particle settles preferably in vertical position with a negative wake behind. The understanding of these processes is essential in technology and engineering and is necessary to improve prediction accuracy of large-scale sedimentation processes and biogeochemical cycles in the ocean involving settling of minerals, marine snow, microplastics, and locomotion of microorganisms.

## Introduction

Effects of rheological properties of non-Newtonian fluids on particle settling dynamics have been extensively studied in the context of applied sciences, while the same issues present in nature have not yet gained much attention, despite evident impact of natural waters rheology on sedimentation processes. The concept that seawater may locally exhibit non-Newtonian properties was presented in the late 1980’s^1^. Further research reported that extracellular polymeric substances (EPSs), known also as exopolymers, which are produced by bacteria and microalgae constitute considerable portion of dissolved organic matter (DOM) in the ocean^2^ and may locally change natural waters into a non-Newtonian liquid. It has been demonstrated that positive correlation exists between seawater viscosity and marine bacterial communities^3^ and seawater viscosity and chlorophyll *a* concentration during algal blooms^4^. Rheological analyses have shown that aqueous solutions of EPSs secreted by marine organisms exhibit elastic properties and higher viscosity compared to the clear water^5^.

Recent studies have addressed the impact of increased seawater viscosity on the motility of microorganisms in the ocean^6^, however, the effect of rheological properties of natural waters modified by the presence of dissolved exopolymers on sedimentation of various particles: minerals, marine snow, larvae, plankton, and microplastic contaminants, remains untouched research problem. Development of such research is necessary to better understand particles settling dynamics in micro-scale as well as to better assess particle settling fluxes, which play significant role in biogeochemical cycles, ocean productivity, and climate^3,7^. Although there is growing interest in exopolymers in industrial applications and process engineering^8^, particle settling dynamics in exopolymer solutions are not adequately addressed. The issues described above are the motivation for research presented in this study.

Previous research covered a wide range of non-Newtonian fluids including variable viscosity inelastic fluids, constant viscosity elastic fluids, variable viscosity elastic fluids, and has shown that rheological properties modify considerably the settling behaviour of particles compared to Newtonian fluid^9,10^.

It has been demonstrated that in non-Newtonian fluids, unlike Newtonian ones, the fluid in the rear of settling sphere may move upwards forming a negative wake. The negative wake has been observed behind spheres in polymer solutions^11^, micelle solutions^12^, colloidal suspensions^13^, and also in the case of bubbles rising in non-Newtonian fluids^14^.

A number of investigations have provided various explanations for physical mechanisms of negative wake^15^. Generally, it has been acknowledged that both viscoelasticity and shear-thinning are necessary for negative wake to appear^9,10^. Otherwise an extended wake with the flow in the direction of particle movement has been identified. The negative flow has been attributed to the opposing extensional stresses and shearing stresses^11^ or to the relation between elasticity and extensional stresses in the rear of a sphere^16^. The appearance of extended wake and negative wake has been explained by the interplay of two viscoelastic forces — the extensional stress downstream of a sphere and the elastic recoil of shear stress outside the wake^17^. The first one is responsible for the extended wake and the other makes the fluid inside the wake to move upwards. Extensibility of a polymer chains is responsible for the mutual ratio between the two abovementioned viscoelastic forces, and the extended wake and negative wake appears for high and low extensibility, respectively. This explanation has been approved in other research^13,15,18^.

Terminal settling velocity is a basic parameter describing the behaviour of particles moving under the influence of gravity in a quiescent fluid. It depends on size, shape, density of a particle, and fluid physical properties. A particle released from rest into a Newtonian fluid accelerates to finally achieve steady terminal velocity when gravitational force is balanced by frictional force and buoyant force. Detailed measurements have revealed that in various non-Newtonian liquids settling velocity does not achieve constant value and that it exhibits fluctuations. An unsteady settling has been observed in hydroxyl propyl guar gels^19,20^, suspension of Laponite^21^, micellar solutions^12,22–24^, fibrous suspensions^19^ and corn starch suspensions^25^. Oscillations of velocity have been explained by the rheological properties of non-Newtonian fluid and its internal structure. There are a few working hypotheses.

Transient settling in corn starch suspensions has been explained by jamming mechanism^25^. The layer of jammed corn starch forms around a sphere and retards the particle. When a sphere slows down the layer relaxes, a drag decreases and the particle accelerates again.

Flow instability and resulting fluctuations of particle settling velocity have been most extensively studied for micellar solutions. It has been postulated that transient settling of a sphere in micellar solutions is the effect of breakdown of entangled micelle network in the wake behind the falling sphere due to growing extensional stress^12^. The authors used image analysis methods to investigate flow around a sphere and associated this instability to filament rupture investigated by rheometric methods. Similar but less precise explanation was provided earlier^20^. It was hypothesised that a wake formed behind a particle may act as a rubber band. After stretching to its limit, the rubber band breaks and the particle accelerates. Then a new wake is formed and the cycle is repeated.

According to another hypothesis, the shear-banding is a physical mechanism for the velocity oscillations of settling particle^21–23^. Formation of flow-induced structures around a particle settling in micellar solution has been postulated in response to the shear^22^. Consequently, effective viscosity and drag increase and a particle decelerates. The sphere accelerates after the break-up of structures. According to another study, flow curve and creep tests have indicated that shear-banding takes place in a laponite suspension^21^. The authors have hypothesized that a layer of liquid with low viscosity forms near a settling sphere as a result of the destruction of solution inner structure, which causes particle acceleration. Then the sphere reaches the other layer of undisturbed fluid with higher viscosity, and decelerates again.

Zhang and Muller^26^ performed study using non-shear-banding micellar solutions to exclude potential impact of shear-banding on flow instability, and they have confirmed that extensional flow in the wake alone may be the source of flow unsteadiness and settling velocity fluctuations. The mechanisms triggering unsteady particle settling in micellar solutions have not been yet fully identified. The impact of other effects such as elastic wave effects and slip on the particle surface have been pointed as potential directions of further studies^26^.

In a low Reynolds (Re) number regime, the orientation of non-spherical particle settling in a viscoelastic fluid is markedly different from the orientation in a Newtonian fluid. This is due to the effect of viscoelasticity which is present in a viscoelastic fluid in addition to inertia and viscosity acting on a particle settling in a Newtonian fluid. Viscoelasticity is responsible for normal stresses acting on a particle. The normal stresses are strongest at two points where fast flow is present and streamlines are crowded^27^. High pressures at these points act in opposite directions on a particle, and form turning couples that create rotation. Produced torque tends to turn a non-spherical particle with the major axis vertical.

In a creeping flow regime (Re ≪ 1, i.e. absent or negligible inertia), a non-spherical particle that has a center of hydrodynamic stress (e.g. disk) settles in a Newtonian fluid with its initial orientation (position in which it was released)^28^. Theoretical analysis presented in Happel and Brenner^28^ has demonstrated that settling does not induce rotation in this flow regime, which is the effect of coupling tensor about the center of hydrodynamic stress equal zero. Conversely, a non-spherical particle settling in a creeping flow regime in a viscoelastic liquid tends to fall with the broadside parallel to gravity^29^. The particle rotates to achieve this orientation as the effect of viscoelastic torque unless the particle has been released in vertical position.

When Re number is larger, i.e. inertia is more significant, turning couples appear at two stagnation points, where high pressure occurs, and form the inertial torque which tends to turn the particle with the major axis horizontal. Consequently, a non-spherical particle assumes horizontal position in a Newtonian fluid. Terminal orientation of a non-spherical particle settling in a viscoelastic fluid is controlled by the ratio between inertial and viscoelastic torques which have opposite signs^10,27,30–32^. Equilibrium particle orientation depends on the net torque and may vary between 0° to 90° depending on the ratio between viscoelastic and inertial effects^32^.

In this study, we address abovementioned aspects of particle settling behaviour to show what non-Newtonian effects may be expected in natural waters with dissolved exopolymers. The aim of this study is to assess how rheological properties of exopolymer aqueous solution affect the settling dynamics of individual solid particle. The objectives of the study are threefold: (1) to evaluate how rheological properties of solutions change with the content of dissolved exopolymer, (2) to assess the effect of exopolymer content in solution on particle settling velocity and drag, (2) to analyse occurrence and character of settling velocity fluctuations. Settling behaviour of particles is quantified and described along with explanation of potential underlying mechanisms.

The objectives are achieved through experimental study comprising rheological measurements of test exopolymer solutions and settling experiments using visualization and particle tracking methods. We studied settling of spheres and disks in aqueous solutions of various content of xanthan gum (XG), a commercial exopolymer applied in environmental experimental studies as a model of EPSs occurring in natural aquatic systems^33^.

Xanthan gum is a water-soluble polysaccharide secreted by a bacterium *Xanthomonas campestris*. It is widely used in food, pharmaceutical, cosmetic, and petroleum industry as a rheology modifier thanks to its unique shear-thinning and viscoelastic properties^34^. Xanthan gum is a polymeric exocellular heteropolysaccharide that forms hydrocolloids in aqueous environment. XG is composed of connected glucose units, that form specific helical conformation with tri-saccharide side chains located along the backbone. There is ongoing debate on the details of XG structure^35^. In ordered state, XG forms network that comprises aggregates made by macromolecules connected by hydrogen bonds and entanglements. In dilute solutions, polymer macromolecules are separated, they entangle when concentration increases^36^, which in consequence amplifies elasticity and shear-thinning effects^37^.

The polymer belongs to negatively charged polyelectrolytes due to the presence of carboxyl groups in the structure. In a deionised water the macromolecule takes an extended conformation due to the electrostatic interactions between negatively charged side chains^38^. This electrostatically driven expansion of the polymer macromolecules in water results in their larger volume and affects the rheological properties of the polymer solution. At higher concentrations the distances between swollen particles become smaller, which intensifies interactions between macromolecules and enhances the rigidity of the physically formed weak polymer gel.

We hope that the results presented in this study will contribute not only to our understanding of the effects of exopolymers in industrial applications but will be also the inspiration and motivation to more intensive research on the impact of rheology of natural waters on sedimentation processes.

## Results and Discussion

### Rheological behaviour of solutions

Time-dependent rheological properties of six aqueous xanthan gum solutions, the same that were used in settling experiments, were measured. Solutions of the following XG content were analysed: 0.25 g/L, 0.50 g/L, 0.75 g/L, 1.00 g/L, 1.25 g/L, and 1.40 g/L. Details on the preparation of solutions is presented in Methods section.

**Figure 1:**
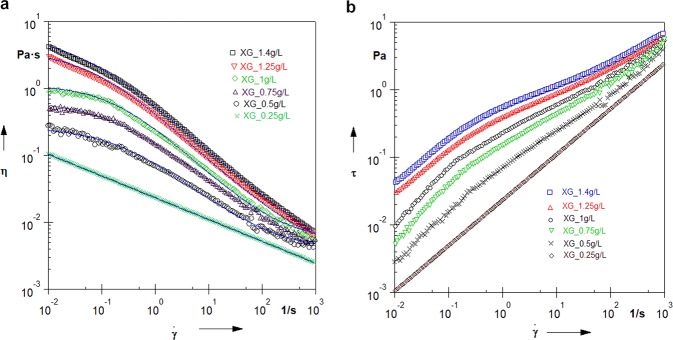
Rheology measurements of investigated xanthan gum solutions. (**a**) Viscosity curves. Solid blue lines represent the fits of experimental data with Cross model. Parameters given in Supplementary Table 1. (**b**) Flow curves.

The viscosity curves of the xanthan gum solutions, recorded at 21 °C in a constant shear rate mode are shown in Fig. 1a. Experimental data were fitted to the Cross equation^39,40^:

1 $$\eta ={\eta }_{\infty }+\frac{{\eta }_{0}-{\eta }_{\infty }}{1+{(B\dot{\gamma })}^{p}}$$

where $\dot{\gamma }$ is the shear rate, *η*_0_ is the zero shear viscosity, corresponding to the viscosity of the solution at extremely small shear rates (lower Newtonian plateau). The coefficient *η*_∞_ is the infinite shear viscosity indicating a viscous behaviour of a liquid at very high shear rates (upper Newtonian plateau). The exponent *p*, known as the (Cross) rate constant, reflects the steepness of the slope of a viscosity curve in the shear-thinning region. *p* = 0 corresponds to clear Newtonian flow, while *p* approaching to 1 indicates the shear-thinning behaviour of the investigated system. The parameter *B* is called consistency (or Cross time constant, as it has a dimension of time). The reciprocal, 1/*B*, indicates an onset of a critical shear rate at which the shear thinning behaviour begins.

Fitting parameters with Cross equation are collected in Supplementary Table 1. All the investigated solutions show shear-thinning behaviour characteristic for polymer solutions with the *η*_0_ viscosity strongly dependent on the xanthan gum concentration, which is well consistent with the results reported earlier on similar systems^36,37,41–43^. Zero shear viscosity of the investigated XG solutions grows exponentially with polymer concentration (Supplementary Fig. S1) due to enhanced entanglement of macromolecules at higher polymer contents. Also, shear-thinning properties, characterized by parameter *p*, increase with XG content in solution (Supplementary Table 1), which is also seen from flow curves presented in Fig. 1b.

**Figure 2:**
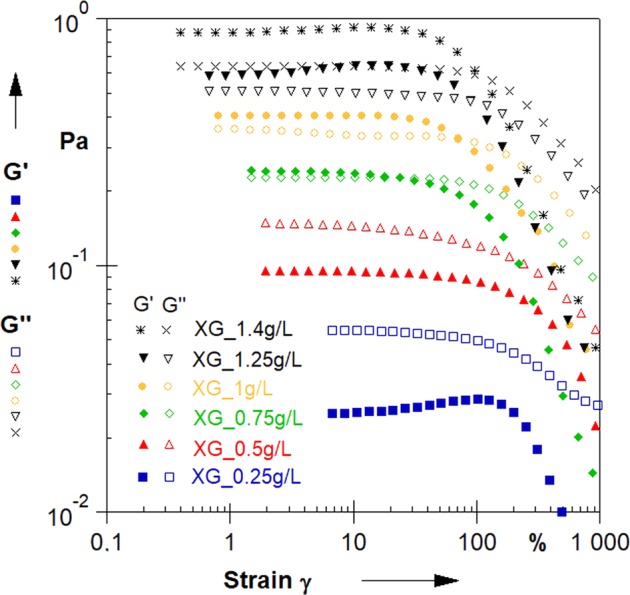
Strain amplitude sweeps for the investigated materials.

Settling of solid particles in polymer solutions is closely related to their viscoelastic behaviour and the possible local jellification preventing fast movement of the rigid body through the system. To reveal the viscoelasticity of our XG solutions and correlate their rheological behaviour with particle settling, we have additionally performed oscillatory tests. Figure 2 shows dependences of the storage modulus (G’) and the loss modulus (G”) on imposed stress amplitude in the range 0.001–10 Pa, obtained at constant frequency 1 Hz for all the investigated materials. Linear viscoelastic (LVE) regime, studied by oscillatory tests, corresponds to the ability of the system to formation of stable gel-like network and provides information about its mechanical properties and susceptibility to deformation. In gel-like materials both the storage modulus G’ and loss modulus G” are independent of strain at low amplitudes, which is revealed by domination of G’ over G”. Moreover, at large strain amplitudes G’ values drop rapidly and are accompanied by great G” enhancement, as expected for gel-like systems. The width of linear viscoelastic range and formation of the microstructure depends on the polymer concentration. It is clearly visible that solutions containing 0.25 g/L and 0.5 g/L of dissolved polymer behave like typical viscoelastic liquids. The solution of 0.75 g/L XG exhibits transient behaviour indicating to possible formation of interacting aggregates, while at higher concentration clear gel-like behaviour is observed. Our observations are consistent with recent literature data^37^, which confirm that transition of XG solution between dilute and semi-dilute state occurs around this concentration.

The parameters of the viscoelastic state, i.e. linear viscoelastic range, maximum deformation before flow point as well as crossover point parameters for the investigated solutions are collected in Table 1. Fluids of XG content below 0.75 g/L flow under any deformation over the whole measured range, therefore for them only the linear viscoelastic range limits were anticipated. Relaxation times for all the investigated materials were estimated on the basis of the frequency sweeps presented in Supplementary Fig. S2. Frequency dependencies of storage modulus (G’) and loss modulus (G”), shown in the figure, were measured for all the solutions at constant stress amplitude (0.05 Pa) taken from the linear viscoelastic range to find the relation between rheological properties of the studied materials and the particle settling experiments.

**Table 1:** Linear viscoelastic (LVE) range and crossover point parameters for investigated solutions.

Xanthan Gum Content [g/L]	LVE Range Related To Shear Stress [Pa]	Max Deformation (G’ = G”) [%]	Crossover Point [Pa]	Flow Point [Pa]	Relaxation Time [s]
1.40	0.484	105.21	0.580/0.237*	0.865	2.517
1.25	0.410	89.10	0.463/0.198*	0.583	1.622
1.00	0.161	73.51	0.463/0.169*	0.335	0.878
0.75	0.102	2.98	0.255/0.127*	0.011	0.519
0.50	0.065	N/A	0.083*	N/A	0.331
0.25	0.043	N/A	0.044*	N/A	0.187

*Indicates values estimated from frequency sweep.

The studied xanthan gum solutions exhibited moderate frequency dependence of their G’ and G” moduli at lower frequencies with distinct crossover points at characteristic frequencies indicating phase transitions. Location of these characteristic points is strongly associated with the solution concentration. The higher concentration of polymer the lower the characteristic frequency, which corresponds to relaxation time for a given system.

In strain-dependent networks, a higher dependence on frequency for the dynamic moduli and smaller differences between the corresponding moduli values is expected. In our case the difference between storage and loss moduli for the investigated materials is rather small, however, it is noticeable for higher concentrations, which gives the indication of gel-like network formation due to the enhanced interactions between macromolecules. The presence of these networks is characteristic to stationary conditions, and when the system deviates from stationary state they flow upon relatively small stresses exhibiting shear-thinning behaviour.

The first normal stress differences N_1_ for investigated materials estimated on the basis of small strain oscillatory measurement are shown in Supplementary Fig. S3. The normal stress difference is positive for all the investigated materials, as expected for solutions of natural polymers (see. e.g Macosko^39^) and increases with the concentration of the polymer. Character of the dependence of N_1_ on the shear strain can be well described with the following equation: N_1_ = *N*_1,0_ + *Aγ*^*n*^, where: N_1,0_ - is a value of N_1_ extrapolated to zero strain, *A* - characterizes consistency of the material under deformation, *n* - exponential factor (Supplementary Fig. S3 and Supplementary Table T6). It is also noticeable that at low strains (up to 3% for the most concentrated fluid), N_1_ values were found to increase linearly with the applied strain, which confirms the elastic properties of the network formed by mutual interactions between negatively charged polymer helices at stationary conditions.

### Characteristics of particle settling dynamics

We studied experimentally settling dynamics of six types of particles (spheres with diameters 1.6 mm and 3.0 mm and four types of thin disks with diameters 1.5 mm, 2 mm, 2.5 mm, and 3 mm, and thickness 0.3 mm), settling freely in six xanthan gum aqueous solutions varying in XG content (concentrations from 0.25 g/L to 1.40 g/L).

Experimental sets are named after experimental conditions, i.e. the concentration of XG, XG _*C*_, where *C* - xanthan gum content in solution [g/L] and the type and diameter of particle, S_*d*_, D_*d*_, where S stands for sphere, D for disk, *d* is particle diameter [mm]. All details on materials and experimental settlings are presented in Methods section.

All particles were settling in transient motion exhibiting fluctuations of settling velocity. The averaged velocity, *U*, was calculated in a similar manner as in other studies^19,26^. Instantaneous settling velocity, *u*_*y*_, measured in a single experimental run was time-averaged. Since several repetitions of each experiment were performed, calculated velocities were next averaged over all repetitions of the experiment to assess *U* (see Methods section for details).

While settling dynamics in a Newtonian fluid are conveniently analysed based on particle Reynolds number, similar relations for non-Newtonian liquid is more ambiguous due to shear-dependent viscosity and elastic effects^9,44^. Additional dimensionless parameters relevant in the problem of sedimentation in non-Newtonian fluid are Deborah number (De) and elasticity number (El). Data collected in this study are described and analysed in terms of these parameters.

Particle Reynolds number for a particle settling in a non-Newtonian fluid (termed also modified Reynolds number) is defined as:

2 $${{\rm{Re}}}_{{\rm{M}}}(\dot{\gamma })=\frac{{\rho }_{f}Ud}{\eta (\dot{\gamma })}$$

where *d* is particle diameter [m], *ρ*_*f*_ is density of fluid [kg/m^3^], $\eta (\dot{\gamma })$ is shear-dependent viscosity [Pa s] calculated with Cross model (Eq. (1)) (parameters in Supplementary Table 1), and $\dot{\gamma }$ is characteristic shear rate induced by a settling particle [1/s] given by $\dot{\gamma }=U/d$^13,23^ or $\dot{\gamma }\mathrm{=2}U/d$^9,45,46^. Characteristic shear rates in this study were evaluated with $\dot{\gamma }=2U/d$ for spheres and $\dot{\gamma }=U/d$ for disks and varied between 0.07 1/s and 42.17 1/s.

Particles were settling in a low Reynolds number regime as indicated in Supplementary Figs S4 and S5 and Re_M_ exceeded unity only for 5 experiments, in which case the significant influence of inertial force on settling dynamics may be expected. Inertial effects revealed in the orientation of disks settling in a solution containing 0.25 g/L xanthan gum for which Re_M_ was around 1, since vertical position of disks was not stable showing some tendency to tilting unless the particle was introduced perfectly vertical. In experiments for which Re_M_ ≪ 1 (i.e. negligible inertia), disks achieved stable vertical orientation, no matter the position of release, which indicates that viscoelastic torque overcame inertial one. These observations are in accordance with other studies on non-spherical particles in viscoelastic fluids^27,29^.

Deborah number^9,10,47,48^, given by Eq. (3), is applied in sedimentation studies to characterize the ratio of the fluid relaxation time, *λ*, and characteristic time of the flow, *t*_*d*_ expressing the residence time of polymer molecule near the settling particle. The characteristic flow time, *t*_*d*_, is evaluated as a reciprocal of characteristic shear rate $\dot{\gamma }$ induced by a settling particle^9,12^ defined above in the text.

3 $${\rm{D}}{\rm{e}}=\frac{\lambda }{{{\rm{t}}}_{{\rm{d}}}}$$

Deborah number is very often used interchangeably with Weissenberg number^9,45,49–53^. However, physical interpretation of these two dimensionless numbers is not the same^10,54^. Weissenberg number is appropriate only when walls of settling tank (boundaries) are far from the settling particle^9^.

Deborah number is presented in sedimentation studies as a parameter characterizing viscoelasticity of a fluid^26^. In light of this statement, decreasing trend of De with the increase of *C* presented in Supplementary Fig. S6 seems to be incorrect, since the viscoelasticity of test solutions increases with the concentration as indicated by increasing relaxation time (Table 1). However, it should be noted that De number in the context of sedimentation does not describe a property of a fluid itself but rather compares the time scale of polymer macromolecules relaxation and time scale of particle settling. D*e* ≫ 1 indicates that a polymer macromolecule sheared by a settling particle is relaxing for a relatively long time after the particle has passed the macromolecule, hence viscoelastic effects are significant in the settling process. Conversely, De < 1 indicates that the relaxation time scale is smaller than the time scale of particle settling, and viscoelastic effects may appear to be negligible in the process. In a shear-thinning viscoelastic fluid, shear-thinning is dominant mechanism affecting particle settling dynamics in a low De number regime^9,12^.

It should be noted that in our study Deborah number is evaluated using relaxation time estimated on the basis of data derived from frequency sweeps of polymer solutions carried out with an imposed small oscillatory shear taken from linear viscoelastic region (which in fact is in the limit of very low shear rates), and elastic effect in the flow past a particle may be not perfectly accounted, which was also pointed by Becker *et al*.^47^. It seems that shear-rate-dependent Deborah number used by Arigo and McKinley^11^ may be more sensible in sedimentation studies, because it better reflects actual elastic effects in the flow induced by a settling particle. Moreover, extensional Deborah number, using extensional fluid properties instead of shear properties, seems to be better suited to characterize interactions between settling particle and flow^12,26^.

Elasticity number^9,11^ does not depend on dynamics of the process and characterizes elastic properties of a fluid, and is defined as:

4 $${\rm{El}}=\frac{{\rm{De}}}{{\rm{Re}}}\mathrm{.}$$

Supplementary Fig. S7 shows that elasticity number increases with XG content as expected, which is in line with results of small strain oscillatory measurements performed on our materials (see Fig. 2 and Supplementary Fig. S3).

### Fluctuating settling velocity

Experimental measurements revealed that a particle settling freely in XG solution does not reach constant terminal velocity, instead settling is unstable. Measurements were taken far enough from the point of particle release (about 0.35 m) to exclude the impact of initial velocity fluctuations that may appear near the point of release^9,12^.

**Figure 3:**
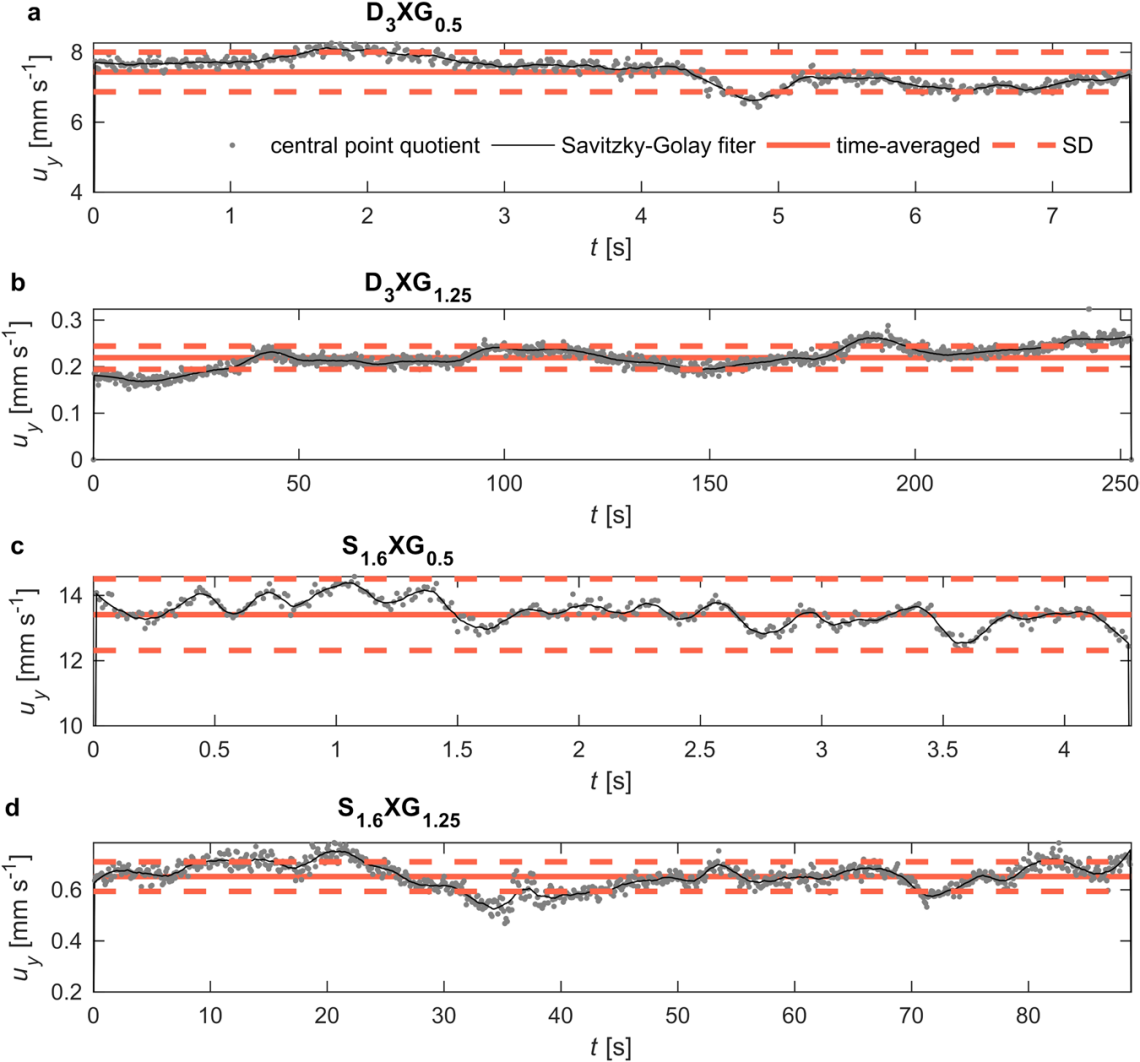
Temporal variability of instantaneous settling velocity *u*_*y*_ of disks (**a**,**b**) and spheres (**c**,**d**) for sample experimental runs. Measured velocity is depicted by grey points and smoothed data by a black line. Time-averaged velocity was evaluated from measured velocity time series for each experimental run. Detailed information on measurements and data analysis is provided in Methods section.

Figure 3 shows the results of temporal variation of instantaneous settling velocity measured for sample experimental runs, where smooth accelerations and decelerations are observed (refer to Methods section for details on instantaneous velocity evaluation). The fluctuations resemble oscillations observed in laponite suspensions^21^, while they are different from fluctuations reported in micellar solutions where pulses of higher velocity have occurred with short acceleration and long deceleration periods^12,22,23^.

Since substantial fluctuations of instantaneous settling velocity, *u*_*y*_, were observed, the strength of fluctuations for each experiment was characterized by the standard deviation (*SD*) from the average settling velocity, *U*. Coefficient of variation, *CV*, which is a statistical measure to assess the extent of variability in relation to the mean, was evaluated to compare the intensity of fluctuations between experiments:

5 $$CV=\frac{SD}{U}$$

Results presented in Supplementary Figs S8 and S9 indicate that fluctuations are mild; *CV* does not exceed 0.1 for the majority of cases. The increase of *CV* with XG content is observed for spheres (Supplementary Fig. S9). However, no such trend is observed in the case of disks. Moreover, unexpectedly high *CV* appears for disks D_1.5_ and D_2_ settling in XG solution of 0.75 g/L. Possible explanation for these high values is the fact that transition of solution between dilute and semi-dilute state occurs around this concentration, which has been revealed by oscillatory tests described above. Macromolecules of different aggregation rates are present in a solution, which may have impact on particle settling velocity, especially in lower shear rate regime. This may explain why such effect has not been observed for spheres; their settling velocity is an order higher than that of disks.

A particle moving freely through the network formed by mutual interactions between negatively charged polymer helices at stationary conditions forces the transient changes in macromolecules conformation, which immediately rearrange back to their lowest energetic state. As a free-falling particle pulls the macromolecule out from its equilibrium position in a physically cross-linked gel, the whole network needs to be reconstructed in its immediate vicinity due to the simultaneous disturbance of electrostatic interactions between charged XG chains. These effects may contribute to fluctuations of particle settling velocities.

**Figure 4:**
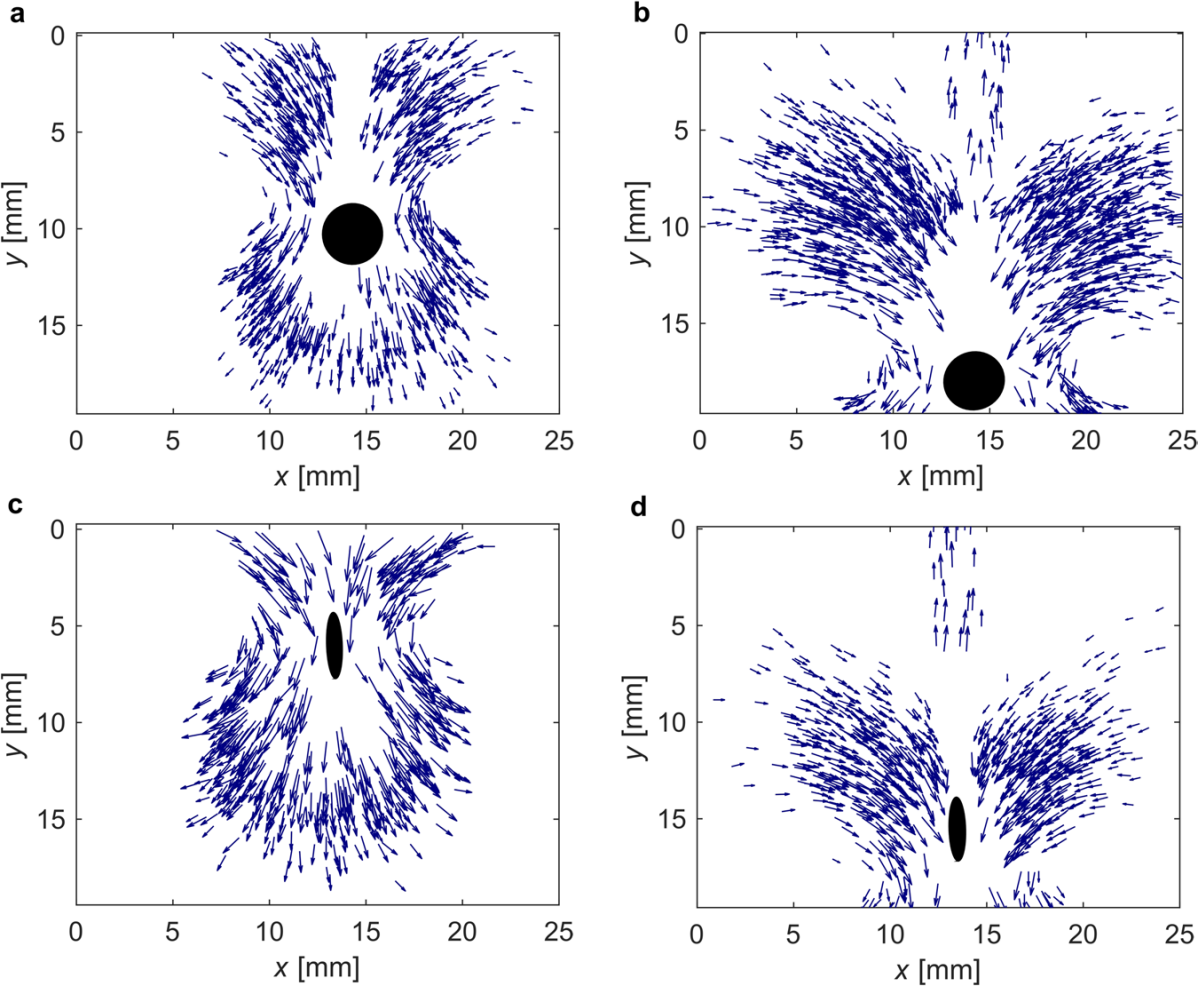
Flow pattern around a settling disk. Results for XG_1.25_ and disk D_3_. A disk is approximated by an ellipse, the velocity field is depicted by arrows (qualitative representation). (**a**) view of the flow ahead disk (front view), (**b**) view of the flow behind disk (front view), (**c**) view of the flow ahead disk (side view), (**d**) view of the flow behind disk (side view).

A negative wake, studied extensively in micellar solutions (see the Introduction) seems to be the prevailing mechanism for settling velocity fluctuations. Particle tracking velocimetry showed that a negative wake appeared behind a sphere settling in XG solution. Moreover, this study has revealed that a negative wake may also appear behind a settling disk and sample results are presented in Fig. 4. The flow pattern around settling disk is similar to that of around a settling sphere; however, is modified by the shape of particle. While the flow forms double-cone downward movement around a sphere^12,13^, the downward flow around a disk has no circular symmetry. It has the cone-like form with two planes of symmetry, instead. Similar to a sphere, a downward flow surrounds an upward negative wake formed behind a disk. Method applied in this work did not allow to study the flow pattern for all experimental conditions, and this remains a subject for further investigations.

We exclude the potential effect of shear-banding on settling unsteadiness, since flow curves (Fig. 1b) do not exhibit plateau region, which may indicate shear-banding. Only recently, it has been reported that shear-banding was observed in concentrated xanthan gum solutions^55^; however, diluted and semi-diluted aqueous xanthan gum solutions, as used in this study, have not been reported to exhibit shear-banding. Further investigation would allow for more detailed studies in this respect.

We observed transient motion of particle in all experiments, representing De numbers in the range (0.95, 12.34) for spheres and (0.11, 1.33) for disks, while research in micellar solutions has reported both steady and unsteady particle settling and extensive studies have been performed to find the criterion for these two modes of motion^12,26^. Generally, a steady motion has been observed for lower settling velocities and the unsteady motion has appeared when this velocity increased^22^. Shear De and extensional De have been applied to correlate the onset of fluctuations and the fluctuations have been tightly connected with the occurrence of negative wake^22,26^. Future studies in xanthan gum solutions could cover wider range of De numbers to verify if steady settling may appear in these fluids.

Since the potential sources of fluctuations in non-Newtonian fluids have not yet been fully explored^26^, other explanations should be verified for xanthan gum solutions. Existing literature suggests that a polymer depleted layer may appear close to the rigid wall of a channel during flow of polymer solution through it. Although the wall-depletion effect, with the depleted layer thickness comparable to the size of the macromolecule, is natural to polymer solutions, it manifests mostly in flow of viscoelastic media through microchannels^56,57^, which is not the case here. As the inner dimensions of the tank used for our settling experiments are large in comparison to possible thickness of the depletion layer (see description of experimental set-up in Methods section), we assume that particle will move freely in an undisturbed fluid. We are aware of the possible formation of a polymer depleted layer close the surface of the settling particle. This could not be confirmed in our experiments, but may be considered in future studies.

### Effects of rheological properties of solutions on the average settling velocity and drag

**Figure 5:**
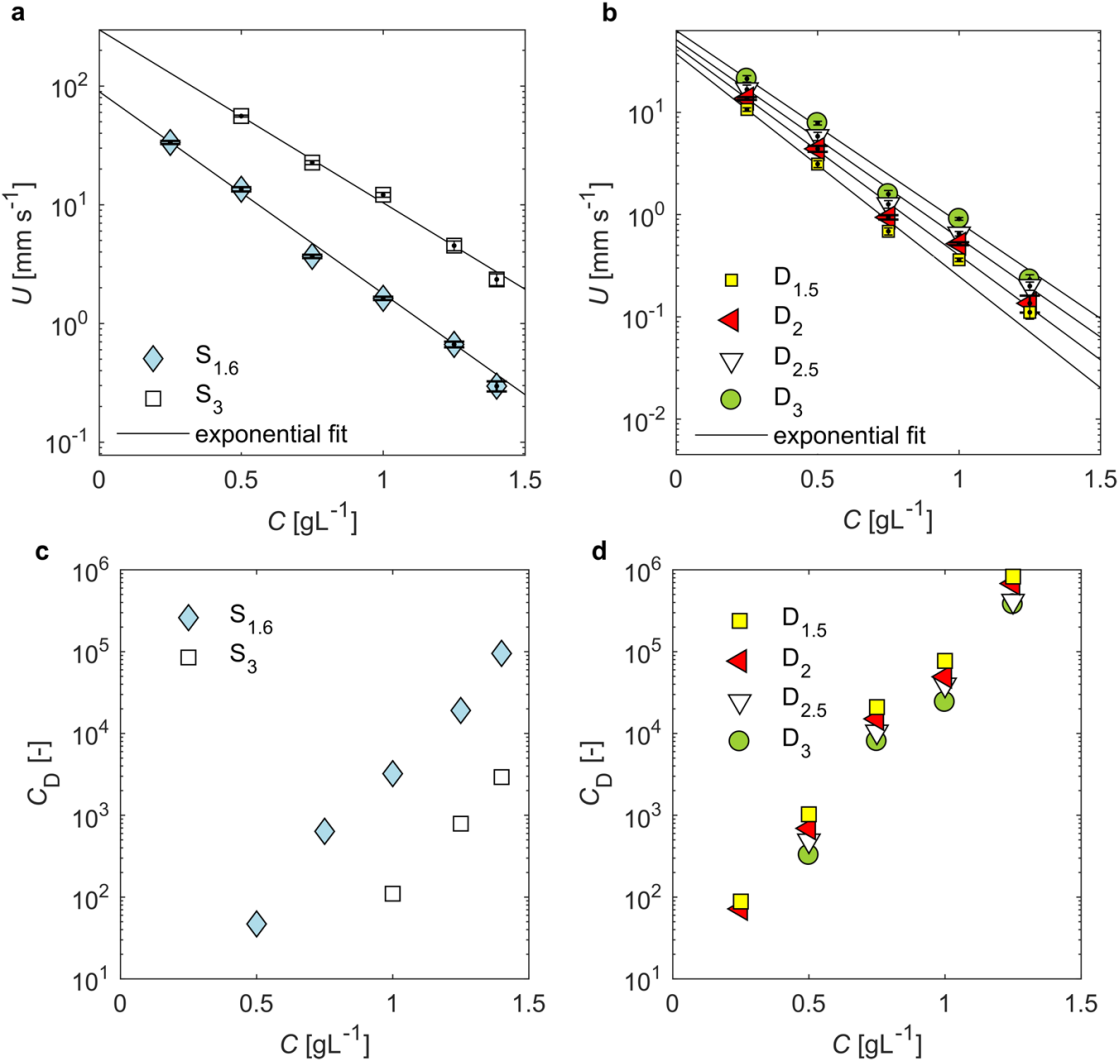
Average settling velocity of a particle *U* (**a**,**b**) and drag *C*_*D*_ (**c**,**d**) as a function of xanthan gum content in solution *C*. Error bars indicate standard deviation, number of experiment repetitions given in Supplementary Table 5. Lines represent the fits of experimental data with exponential model, parameters shown in Supplementary Table 2. *C*_*D*_ was evaluated with Eq. (6) only for experiments where ${{\rm{Re}}}_{{\rm{M}}}(\dot{\gamma }) < 1$.

Results presented in Fig. 5a,b show that average settling velocity, *U*, decreases with the concentration of XG in solution for both disks and spheres. Similar trends have been observed in other non-Newtonian liquids^19,23^. An exponential function was fitted to the data; the fits are presented in Fig. 5a,b and model parameters are given in Supplementary Table 2. Some discrepancies are observed for D_1.5_ and D_2_ compared to disks with larger diameters for concentrations above 0.75 g/L. This could be explained by the influence of elastic effects revealed in oscillatory tests, which may affect settling of particles in low shear regimes.

Formula for creeping flow conditions was applied for experiments where R*e*_*M*_ < 1 to evaluate drag coefficient *C*_*D*_^28^:

6 $${C}_{D}=\frac{gV({\rho }_{p}-{\rho }_{f})}{0.5{\rho }_{f}{U}^{2}A}$$

where *g* – acceleration due to gravity [m/s^2^], *V* – volume of settling particle [m^3^], *A* – projected area of particle normal to flow [m^2^], *ρ*_*p*_ – density of settling particle [kg/m^3^], *ρ*_*f*_ – density of fluid [kg/m^3^].

Results show that the drag coefficient *C*_*D*_ increases with the concentration of solution (Fig. 5c,d), which is in agreement with the decrease in settling velocity shown in Fig. 5a,b. Viscosity is responsible for drag in a creeping flow regime, and the increase in *C*_*D*_ and decrease in settling velocity with concentration of solution is explained by the increase of apparent viscosity with xanthan gum content (Fig. 1a). Drag enhancement is triggered by changes in the structure of solutions with the content of XG, that is, increasing number of entanglements and stronger electrostatic interactions between XG macromolecules and stiffening of polymer network, which oppose downward movement of particle.

We found that the settling velocity of disks at particular concentration of XG solution is positively related to the dimensions of a particle (Supplementary Fig. S10). Similar findings have been reported for other non-Newtonian fluid^19^. The results are well fitted with an exponential function and parameters are given in Supplementary Table 3.

Newtonian drag correction coefficient has been vastly analysed in literature to show how a drag exerted on a particle settling in non-Newtonian fluid changes relative to the theoretical Newtonian drag acting on a particle settling in zero-shear viscosity conditions. This coefficient accounts for elastic and shear-thinning effects on drag^48^. Two equivalent types of drag correction coefficient have been used. The first one, *X*_*e*_, is based directly on drag coefficients^9,49,53^:

7 $${X}_{e}=\frac{{C}_{D}}{{C}_{DS}}=\frac{{C}_{D}{{\rm{Re}}}_{0}}{24}$$

where *C*_*D*_ is defined by Eq. (6) and *C*_*DS*_ is drag coefficient evaluated for Stokes flow^28^ in zero-shear regime corresponding to Reynolds number:

8 $${{\rm{R}}{\rm{e}}}_{0}=\frac{{\rho }_{f}{U}_{S}d}{{\eta }_{0}}$$

where *U*_*S*_ is terminal settling velocity of a sphere in Stokes flow:

9 $${U}_{S}=\frac{g{d}^{2}({\rho }_{p}-{\rho }_{f})}{18{\eta }_{0}}.$$

The second drag correction factor, *K*, is simply a relation between *U*_*S*_ and particle settling velocity in a non-Newtonian fluid, *U*^12,23,48,51^:

10 $$K=\frac{{U}_{S}}{U}\mathrm{.}$$

Other studies considered the effect of walls proximity for *d*/*D* (where *D* is a diameter of cylindrical tank or inner dimension of rectangular tank) ranging from 0.05 to 0.15^51^, *d*/*D* = 0.0625, *d*/*D* = 0.125^12^, and *d*/*D* = 0.067^23^. In this study, the maximum *d*/*D* ratio was 0.05 and is considered negligible.

Previous studies presented a few patterns of *K*(De) relationship depending on the rheological properties of a fluid^12,49,51,53^. Various mechanisms have been indicated to affect the trend of relationship between *K* and De including extensional flow in the region of stagnation points, solvent and solute molecular weights, and shear-thinning of the fluid^9^. These factors are individual for each non-Newtonian fluid and some of them have been summarised in Mendoza-Fuentes *et al*.^51^. Decreasing trend in the relation between *K* and De is usually observed at low Deborah numbers, which is explained by the effect of shear-thinning^12^. As was described above in the text, the temporal scale of elastic effects is too small compared to the time scale of particle motion to affect settling process. The effect of elasticity may be observed for higher De numbers, as has been reported for micellar solutions where *K* has increased with De, which has been attributed to extensional flow effects^12^.

**Figure 6:**
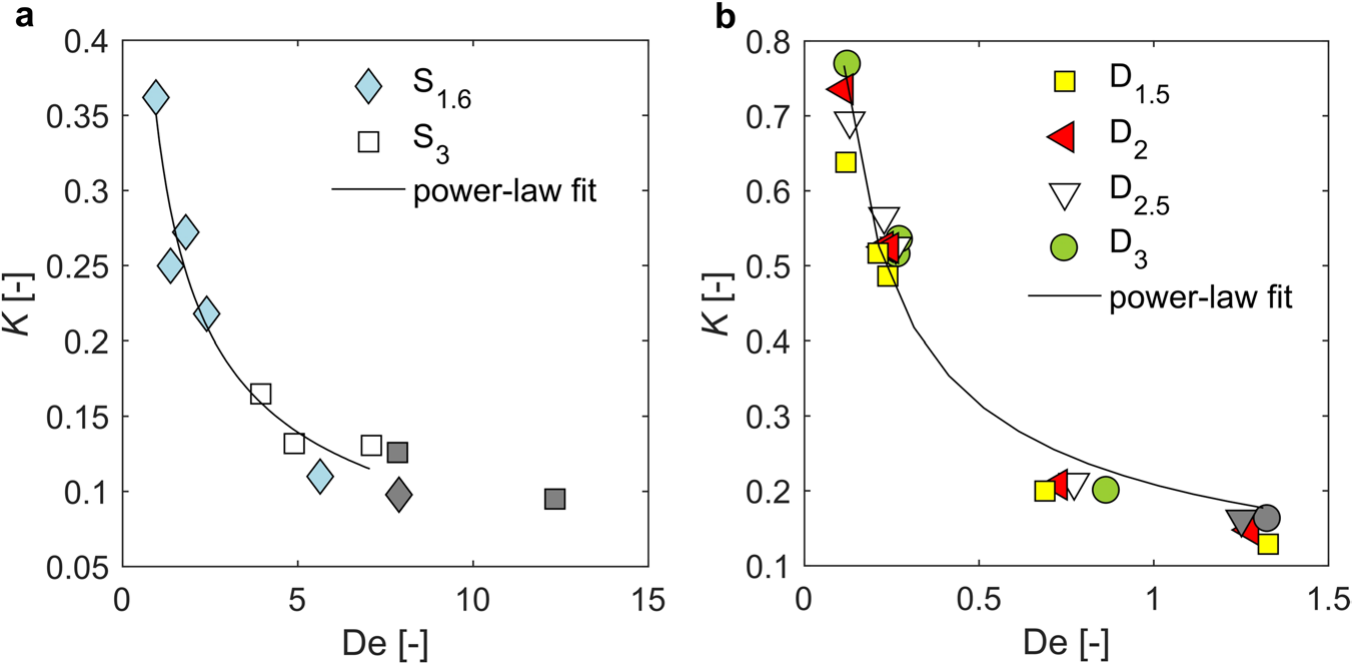
Drag coefficient *K* as a function of Deborah number (**a**), for spheres and (**b**), for disks. Grey symbols denote results for ${{\rm{Re}}}_{{\rm{M}}}(\dot{\gamma }) > 1$. Power function was fitted only to data with ${{\rm{Re}}}_{{\rm{M}}}(\dot{\gamma }) < 1$, model parameters shown in Supplementary Table 4.

In this study, drag correction factor, *K*, was calculated using Eq. (10) for spheres and disks. The settling velocity in a Newtonian flow regime, *U*_*S*_, for disks was related to the terminal settling velocity of equivalent sphere, and *d* was taken as a diameter of equivalent volume sphere. *K* values for all experiments are less than 1, which means that a particle falls faster in a considered non-Newtonian fluid than in a reference Newtonian fluid with viscosity equal to zero-shear viscosity, which reflects the impact of shear-thinning effect of the fluid on the drag reduction. The results are presented as a function of Deborah number in Fig. 6. *K* decreases as De increases in the considered range of De numbers – (0.11, 1.33) for disks and (0.95, 12.34) for spheres. The results are well fitted with a power-law function and parameters are given in Supplementary Table 4. This trend is in line with similar studies in viscoelastic shear-thinning fluids^51^. Since no increase in *K* with De is observed, it could be stated that drag in considered conditions is affected by the fluid shear-thinning properties.

This study is not exhaustive, and further detailed studies on rheological aspects of sedimentation in exopolymer solutions are necessary, as have been performed for other non-Newtonian liquids, e.g. micellar solutions. Potential directions for future studies are research on the occurrence and sources of settling velocity fluctuations, flow structure around settling particle and the onset for negative wake. Wider particle size range and density range could be used in future studies to obtain larger variability of Re and De numbers. The presence of charged functionalities on the polymer backbone may affect the settling behaviour of the particles due to the possible interaction of negatively charged macromolecules and particle surface charges. These phenomena have not been studied so far. Moreover, to model more realistic natural conditions, ionic solutions of exopolymers should be investigated to account for charge screening effect on rheological properties of xanthan gum solution^38^.

## Methods

### Preparation of xanthan gum aqueous solutions

Commercial powdered xanthan gum (XG) was used to prepare six XG solutions with concentrations 0.25 g/L, 0.50 g/L, 0.75 g/L, 1.00 g/L, 1.25 g/L, and 1.40 g/L. To get the desired concentrations of xanthan gum in water, the aliquots of XG powder were added to containers filled with distilled water and kept quiescent for three days to hydrate the polymer. Then the solutions were mixed to get homogeneous solution. Given the small mass of added polymer, the density of solutions was almost the same as for water. Solutions were prepared in Hydrodynamic Micromodels Laboratory, Institute of Geophysics, Polish Academy of Sciences where they were used in settling experiments. A sample of each solution was taken for rheology measurements.

### Rheology measurements

Rheological studies were carried out at Department of Inorganic Chemistry, Faculty of Chemistry, Warsaw University of Technology using Physica MCR 301 rheometer equipped with coaxial cylinder measuring geometry of 0.714 mm gap. Measurements were carried out at constant temperature 21 ± 0.02 °C, to match temperature during settling experiments, controlled by a built-in precise Peltier device. Rheological tests in a controlled shear rate (CSR) mode were performed to reveal the shear-thinning behaviour of xanthan gum solutions at the shear flow conditions.

Viscoelastic properties of the xanthan gum solutions were examined with a small-strain amplitude oscillatory experiments. Characteristic relaxation times of the XG solutions were estimated on the basis of frequency dependencies of storage (G’) and loss (G”) moduli recorded at constant stress amplitude (0.05 Pa) in the frequency range 0.01–100 rad/s.

First normal stress difference values for the investigated materials were evaluated on the basis of small-strain amplitude oscillatory experiments using Lodge-Meissner relation that holds well also for diluted and semi-diluted polymer solutions^39,58,59^.

### Settling experiments

Particle settling experiments were performed in Hydrodynamic Micromodels Laboratory, Institute of Geophysics, Polish Academy of Sciences. Six types of particles were used in experiments. Spheres of two diameters 3.00 mm and 1.588 mm were made of POM (polyoxymethylene) with a density of 1.41 g/cm^3^. Disks were made of PCV (polyvinyl chloride) with a density of 1.43 g/cm^3^. All disks were 0.3 mm thick and four diameters: 1.5 mm, 2.0 mm, 2.5 mm, and 3.0 mm, were considered.

Experiments were carried out in a tank with interior dimensions 0.060 m × 0.060 m and a height of 0.500 m. The experimental set-up was similar to one used in previous settling experiments performed in the laboratory^60^ and is presented in Supplementary Fig. S11. Here, only necessary details or modifications of experimental set-up are described.

The tank was filled with homogeneous aqueous solution of xanthan gum. Temperature of liquid was controlled and varied between 20 °C and 22 °C. Particles were released into the liquid column and measurements of their free settling were carried out. In one experiment, settling of several particles of the same type was measured (that is, there were several experimental runs) to collect substantial data sample and ensure the repeatability of measurements. Particles were released one by one every 5–10 minutes to ensure that the solution had relaxed. Supplementary Table 5 reports that each experiment comprised from 6 to 13 runs, which is larger data sample than in similar studies, where 3 or 4 particles were investigated^19,23^. Some types of particles were not examined for extreme concentrations (indicated by zero in Supplementary Table 5). The limitations were too high settling velocities for accurate measurements or visibility problems due to turbidity of the highest concentrated solutions.

A particle was released beneath the liquid surface in the centre of the tank. Spherical particles were released gently by a pincette into a vertical tube of 4-mm diameter with the end immersed about 20 mm below the surface to prevent the particle from lateral movement. A Plexiglas plate was installed vertically in the middle of the tank to facilitate precise release of disks. The plate was immersed about 20 mm below the liquid surface. Disks were immersed in vertical position by a pincette so that the particle touched its broadside to the surface. Then, disk slid on the plate until it was released from the wall and started to settle freely.

Shadowgraph method was applied to visualize settling particles, a backlight was placed behind the tank and particle shadow was recorded in a field of view 77 mm × 62 mm using the digital camera equipped with macro lenses (Supplementary Fig. S11). The particle was recorded at an acquisition rate varying from 1 to 60 fps (frames per second) which was adjusted to settling velocity. The centre of the field of view was positioned 0.35 m below the liquid surface to ensure that a particle achieved terminal settling velocity and residual effects of particle release disappeared.

### Evaluation of instantaneous and average settling velocity

Instantaneous particle settling velocity, *u*_*y*_, was evaluated from the sequence of recorded images using Particle Tracking Velocimetry method. First, the position and orientation of the particle in each image was assessed by image analysis methods described in previous study^60^. Then, knowing a time step between consecutive frames, the particle time-resolved position was evaluated. Finally, the Savitzky-Golay filter was applied to fit the position data to a moving-average cubic polynomial. A central-point difference quotient was applied to evaluate the instantaneous settling velocity. The central-point difference quotient was additionally applied directly to the raw position and time step data to compare this evaluation with the results of the Savitzky-Golay filtering (see Fig. 3).

Particle tracking measurements provided time series of instantaneous settling velocity, *u*_*y*_(*t*) for each experimental run so that dataset for each experiment comprised several time series, one for each experimental run. Time-averaged values of settling velocity, ${\bar{u}}_{y}$, were evaluated for each experimental run. Repeatability of measurements was verified by comparing time-averaged velocity values. No more than three runs of each experiment were excluded from further analysis based on two standard deviations threshold. They were runs where particle probably swerved from the centreline of the tank. Such drift causes misestimating of settling velocity, since the distance between the camera and a particle is a critical parameter in velocity evaluation by image analysis methods. Average settling velocity for each experiment, *U*, represents the average over ${\bar{u}}_{y}$ for selected experimental runs, i.e. repetitions of an experiment.

### Flow pattern analysis – particle tracking velocimetry

Particle Tracking Velocimetry (PTV) method was used to estimate a velocity vector field around a settling particle. The method applied in this study enabled qualitative identification of the flow pattern around settling particle.

A xanthan gum solution was seeded with powdered Al_2_O_3_, which suspended into the liquid and followed its movement. The seeded solution was poured into 0.020 m - thick and 0.250 m - high settling tank. A backlight was used to visualise seeding particles. Low concentration of seeding particles and small lateral dimension of the tank enabled identification of individual seed particles by visualisation methods. The same camera was used as in the settling experiment, but additional extension tubes (16 mm) were applied to obtain field of view 25 mm × 19 mm with one pixel corresponding to 9.65 *μ*m.

Individual seed particles and their centroids were identified in a sequence of multiple images. Multi-frame tracking method was used to identify corresponding particles in a few consecutive images by using the nearest neighbour algorithm. The Savitzky-Golay filter was applied to fit the positions of particle centroid on multiple images to moving-average cubic polynomial. A central-point difference quotient was applied to evaluate horizontal and vertical components of instantaneous velocities. Next, erroneous vectors were identified and discarded.

## Supplementary information

Supplementary Materials

## Data Availability

Correspondence and requests for materials should be addressed to Magdalena Mrokowska (m.mrokowska@igf.edu.pl).
