# Supplementary material for: Viscoelastic and shear-thinning effects of aqueous exopolymer solution on disk and sphere settling
Source: Sci Rep. 2019 May 27;9:7897. doi: 10.1038/s41598-019-44233-z (PMC6536512; doi:10.1038/s41598-019-44233-z)
Supplement: Supplementary file 1 — Supplementary Materials [file 41598_2019_44233_MOESM1_ESM.pdf]

Supplementary Materials

Viscoelastic and shear-thinning effects of aqueous exopolymer solution on disk and sphere settling

Magdalena M. Mrokowska[1]* and AnnaKrztoń-Maziopa[2]

[1*] Institute of Geophysics Polish Academy of Sciences, Ks. Janusza 64, 01-452 Warsaw, Poland
e-mail: m.mrokowska@igf.edu.pl

[2] Warsaw University of Technology, Faculty of Chemistry, Noakowskiego St. 3, 00-664 Warsaw, Poland

Supplementary Table T1. Parameters of fits the experimental data with Cross model.

xanthan gum content [g/L]	zero shear viscosity η_0 [Pa s]	infinite shear viscosity η_∞ [Pa s]	*p* [-]	*B* [s] ^{p}
1.4	5.216	6.38 10^{-3}	0.697	8.856
1.25	3.383	6.11 10^{-3}	0.681	7.799
1.0	1.276	5.32 10^{-3}	0.672	4.578
0.75	0.708	4.13 10^{-3}	0.629	3.774
0.5	0.381	3.75 10^{-3}	0.529	4.691
0.25	0.173	2.46 10^{-3}	0.449	6.867

Supplementary Table T2. Parameters of exponential model $U = a \exp(b\ C)$ and goodness of fit. U – average settling velocity, C – concentration of xanthan gum solution, a, b – model parameters. Results shown in Fig. 5a,b.

Particle	*a*	*b*	R2	RMSE
$S_{1.6}$	89.61	-3.918	0.9977	0.7047
S_3	297.6	-3.355	0.9973	1.307
$D_{1.5}$	37.31	-5.012	0.9993	0.1336
D_2	44.33	-4.708	0.9986	0.2427
$D_{2.5}$	51.17	-4.462	0.9979	0.3694
D_3	62.94	-4.321	0.9964	0.6135

Supplementary Table T3. Parameters of exponential model $U = a \exp(b\, d)$ and goodness of fit. U – average settling velocity, C – concentration of xanthan gum solution, d – particle diameter, a, b – model parameters. Results shown in Supplementary Fig 10.

C [g/L]	a	b	R2	RMSE
0.25	5.4050	0.4558	0.9988	0.1895
0.50	1.3000	0.5996	0.9989	0.0811
0.75	0.3166	0.5409	0.9944	0.0357
1.00	0.1510	0.5957	0.9940	0.0219
1.25	0.05276	0.5018	0.9636	0.0131

Supplementary Table T4. Parameters of power-law model $K = a\, \mathrm{De}^b$ and goodness of fit. K – drag correction factor, De – Deborah number, a, b – model parameters. Results shown in Fig. 6.

Particle type	a	b	R2	RMSE
Spheres	0.3417	-0.5575	0.9428	0.02244
Disks	0.2085	-0.5988	0.9414	0.05274

Supplementary Table T5. Experimental conditions, type of particles and number of settling experiments repetitions. XG_C indicates the type of solution, where C – xanthan gum concentration in solution [g/L]; D_d, S_d indicate type of particle where S stands for sphere, D for disk, d – particle diameter [mm].

	S_3	$S_{1.6}$	D_3	$D_{2.5}$	D_2	$D_{1.5}$
$XG_{0.25}$	0	12	6	12	13	10
$XG_{0.5}$	9	12	9	10	10	11
$XG_{0.75}$	12	11	10	13	10	11
XG_1	14	10	10	11	11	10
$XG_{1.25}$	12	11	11	12	10	11
$XG_{1.4}$	10	8	0	0	0	0

Supplementary Table T6. Parameters of fits the experimental data with $N_1 = N_{1,0} + A\gamma^n$ equation, and a goodness of fit. Results presented in Supplementary Figure S3.

xanthan gum content [g/L]	$N_{1,0}$ [Pa]	*A*	*n*	R^2
1.4	$8.065 \cdot 10^{-7}$	1.019	1.986	0.9775
1.25	$2.300 \cdot 10^{-7}$	0.7252	1.983	0.9940
1.0	$6.781 \cdot 10^{-8}$	0.4740	1.973	0.9790
0.75	$4.012 \cdot 10^{-8}$	0.2907	1.965	0.9883
0.5	$2.771 \cdot 10^{-8}$	0.1522	1.933	0.9919
0.25	$1.567 \cdot 10^{-8}$	0.0499	1.951	0.9935

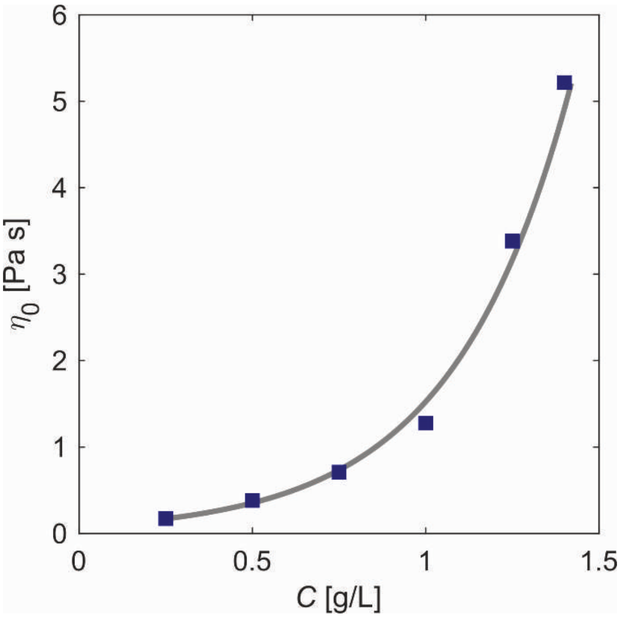

Supplementary Figure S1. Zero shear viscosity dependence on xanthan gum concentration. Line represents the fit of experimental data with exponential model $\eta_0 = a \exp(b\,C)$, a = 0.0819, b = 2.9241, R^2 = 0.9944.

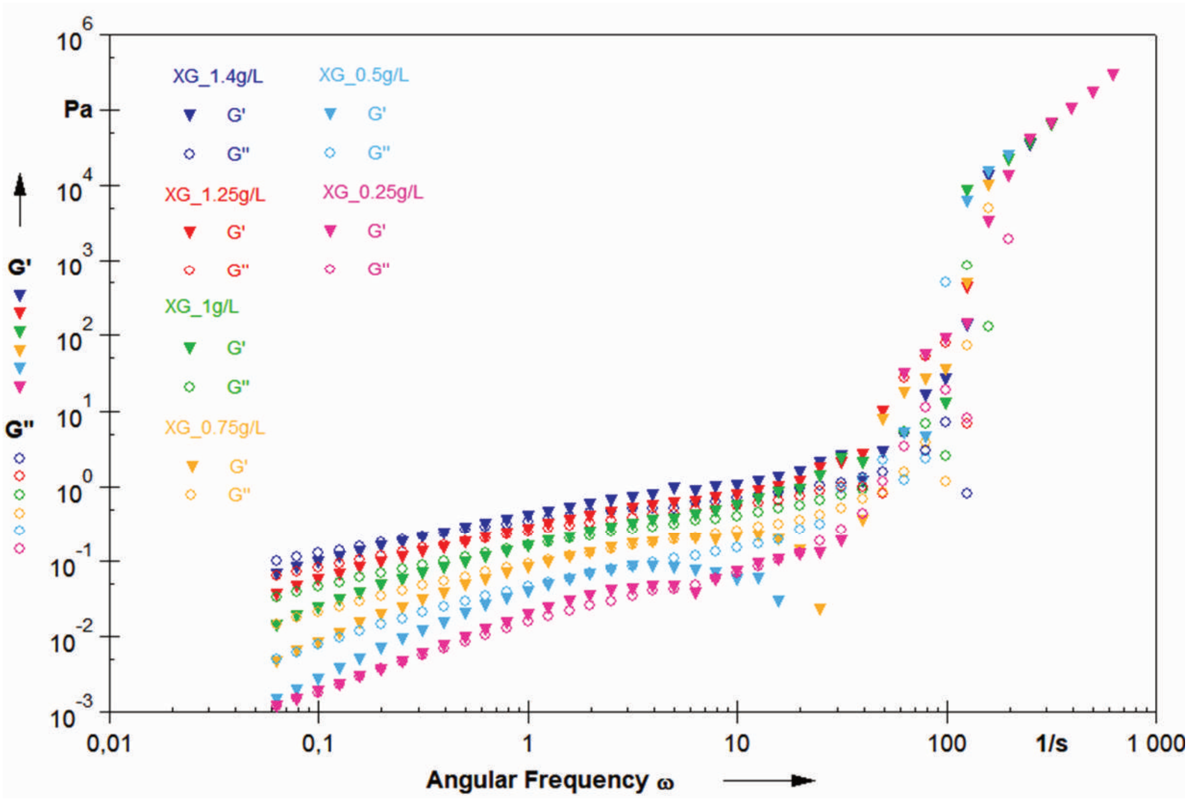

Supplementary Figure S2. Frequency sweeps for the investigated materials.

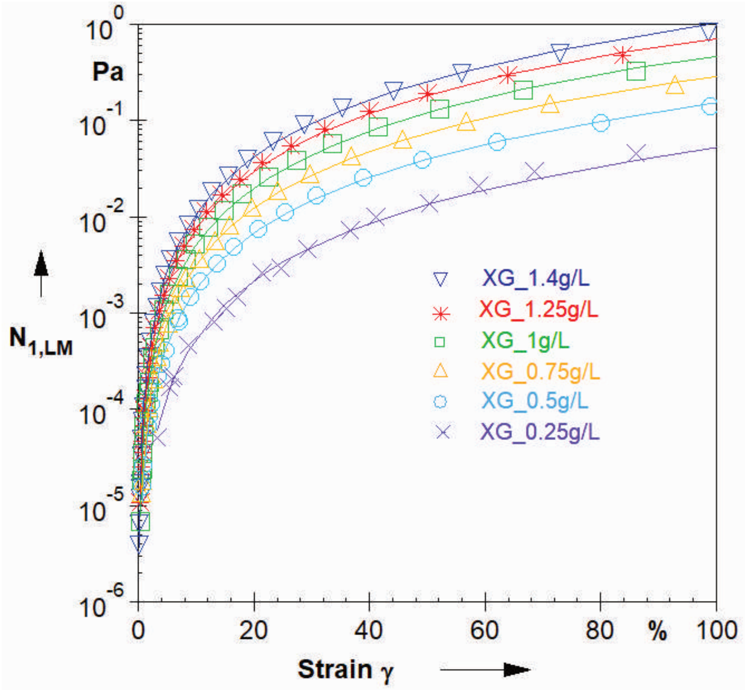

Supplementary Figure S3. Dependence of first normal stress difference (N1) on shear strain for the investigated materials. Solid lines represent the curves fitted to $N_1 = N_{1,0} + A\gamma^n$. Fitting parameters are collected in Supplementary Table T6.

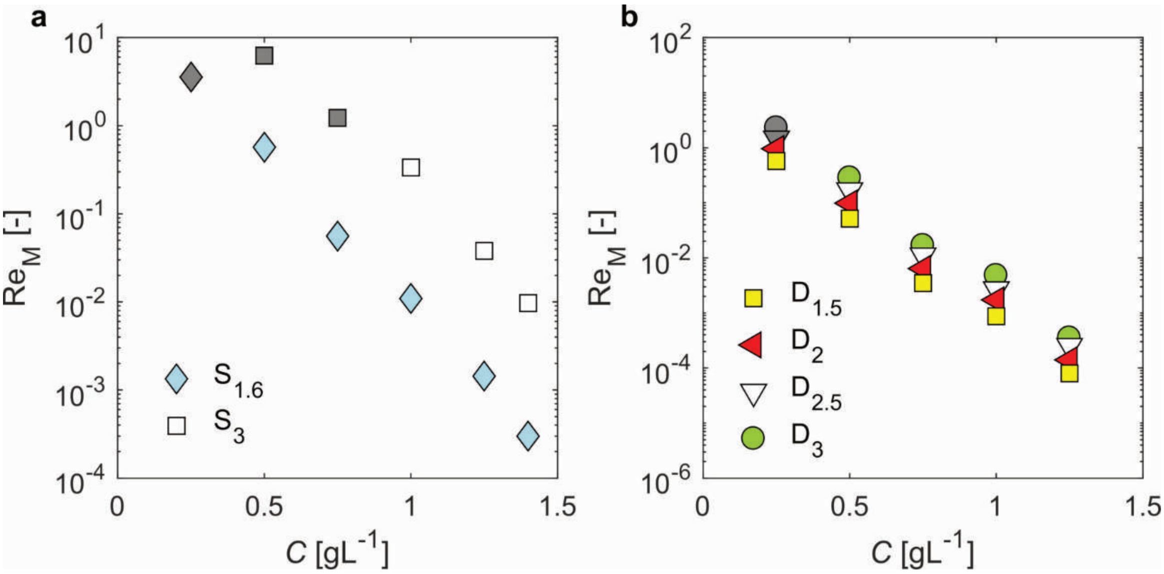

Supplementary Figure S4. Variability of modified Reynolds number Re_M (evaluated with Eq. 2) with the xanthan gum content in solution C. **a,** Results for spheres, **b,** Results for disks. Grey symbols denote results for $Re(\dot{\gamma}) > 1$.

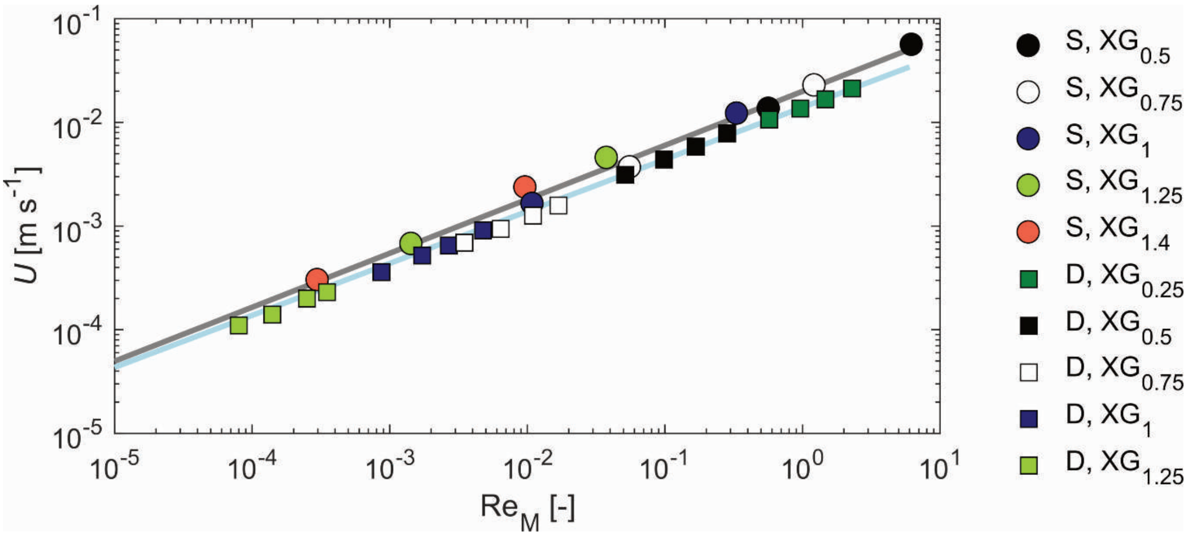

Supplementary Figure S5. Relation between averaged settling velocity U and modified Reynolds number Re_M. Solid lines indicate power-law fitting.

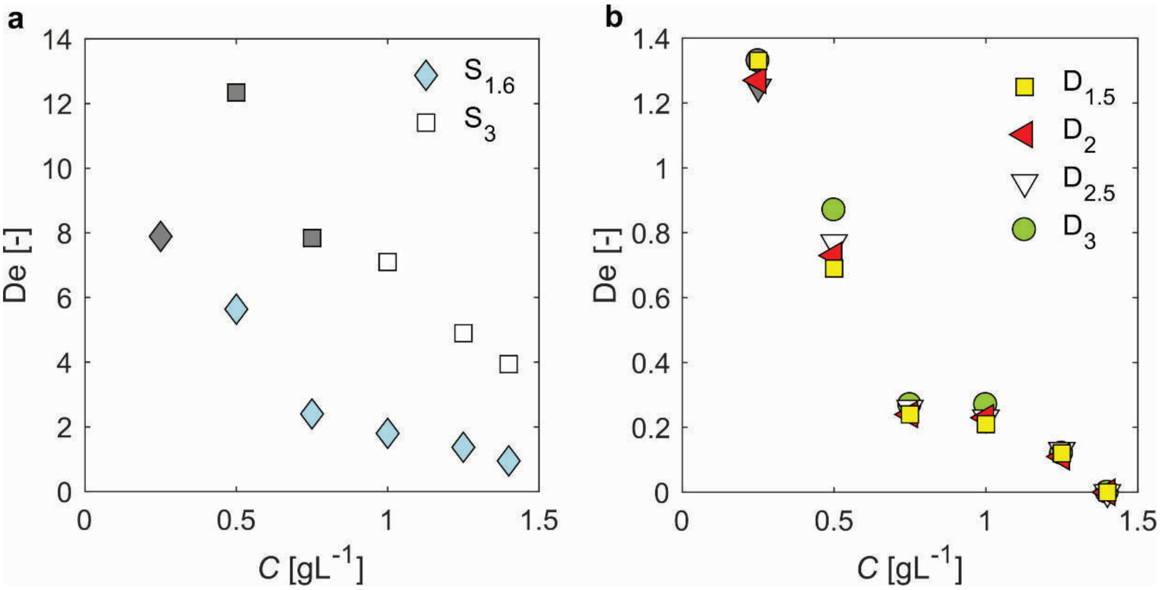

Supplementary Figure S6. Variability of Deborah number De with the xanthan gum content in solution *C*. **a,** Results for spheres, **b,** Results for disks.

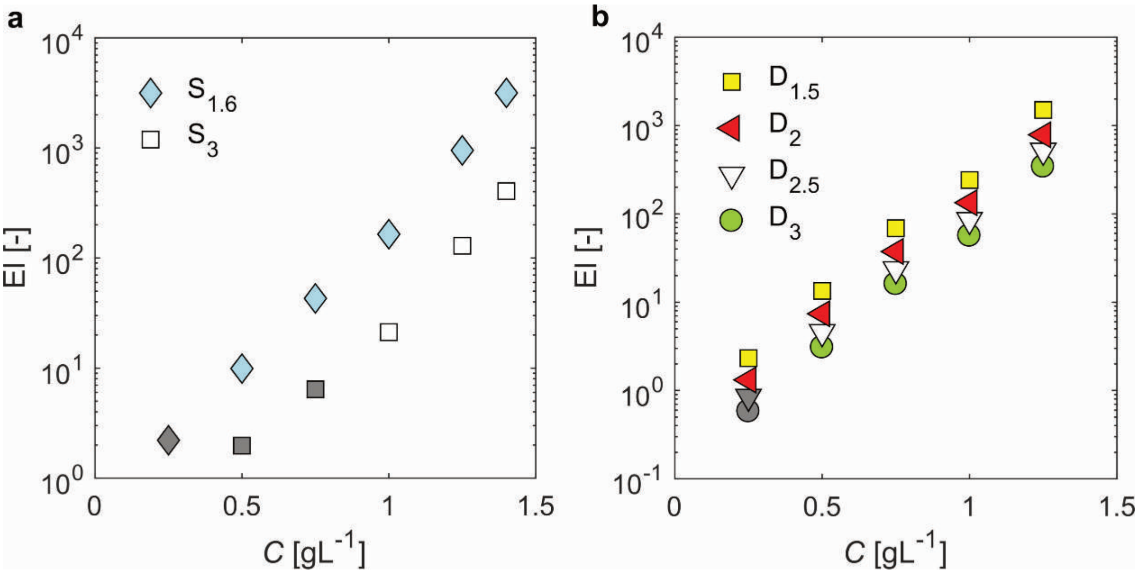

Supplementary Figure S7. Variability of Elasticity number El with the xanthan gum content in solution *C*. **a,** Results for spheres, **b,** Results for disks.

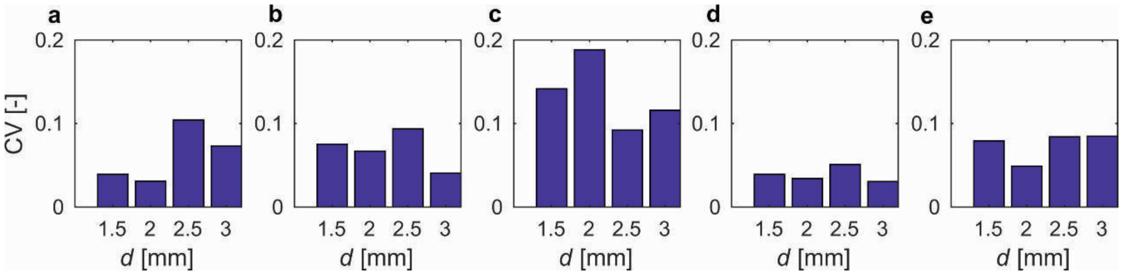

Supplementary Figure S8. Coefficient of variation (CV) for instantaneous settling velocity of disks in xanthan gum solutions of various concentrations (*C*), *d* denotes particle diameter.
a, *C* = 0.25 g/L, **b,** *C* = 0.50 g/L, **c,** *C* = 0.75 g/L, **d,** *C* = 1.00 g/L, **e,** *C* = 1.25 g/L.

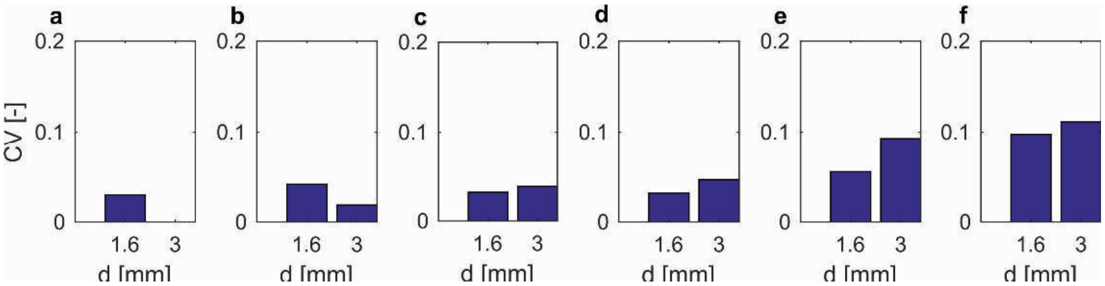

Supplementary Figure S9. Coefficient of variation (CV) for instantaneous settling velocity of spheres in xanthan gum solutions of various concentrations (*C*), *d* denotes particle diameter. **a,** *C* = 0.25 g/L, **b,** *C* = 0.50 g/L, **c,** *C* = 0.75 g/L, **d,** *C* = 1.00 g/L, **e,** *C* = 1.25 g/L, **f,** *C* = 1.4 g/L.

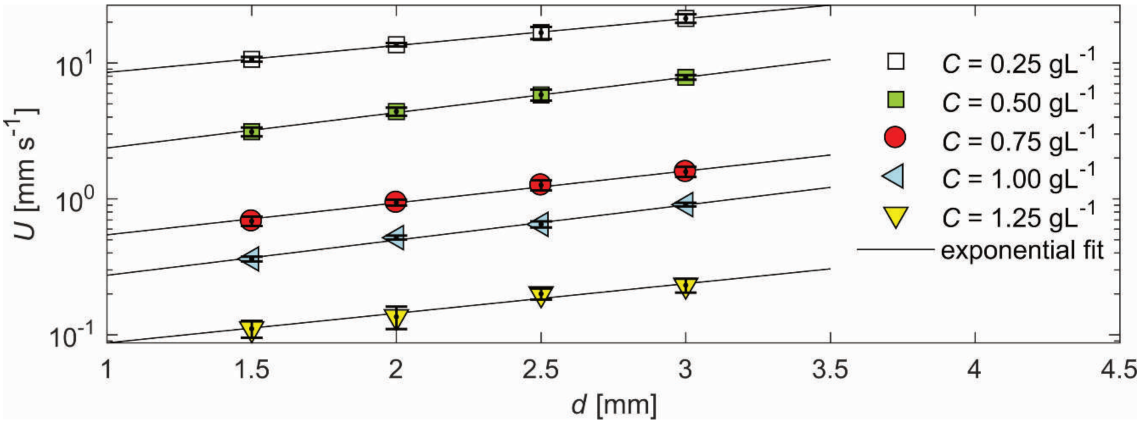

Supplementary Figure 10. Average settling velocity of a disk as a function of particle diameter for different concentrations of xanthan gum solution. Error bars indicate standard deviation. Lines represent the fits of experimental data with exponential model, parameters shown in Supplementary Table 3.

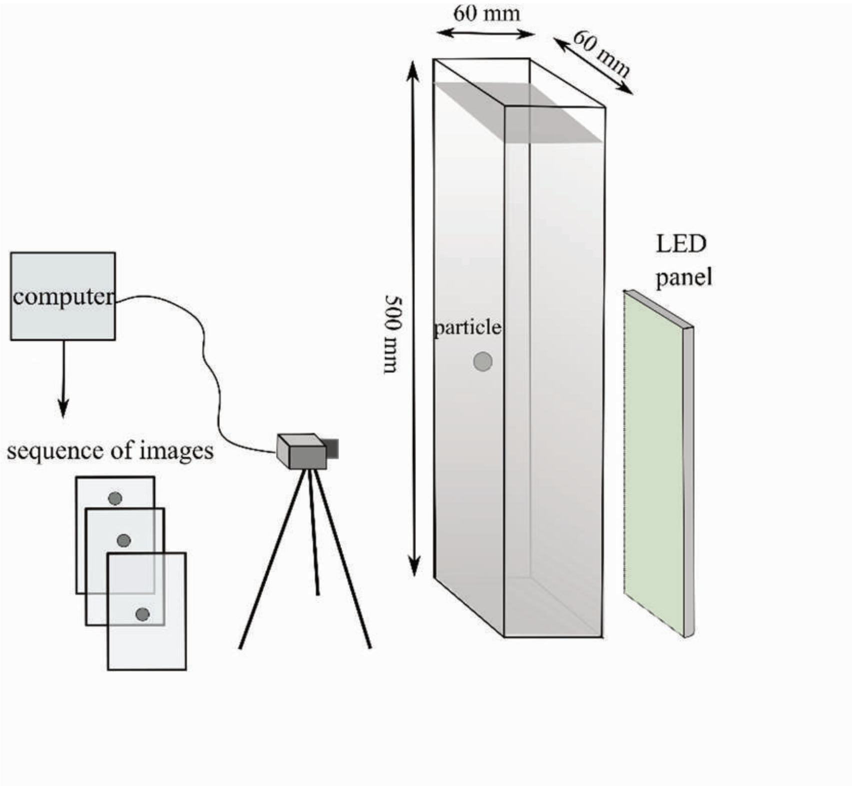

Supplementary Figure S11. Schematic of experimental set-up (settling experiment), not to scale.
